# Ameliorative Effect of Tocotrienols on Perimenopausal-Associated Osteoporosis—A Review

**DOI:** 10.3390/antiox11112179

**Published:** 2022-11-03

**Authors:** Gengfan Liang, Audrey Siew Foong Kow, Chau Ling Tham, Yu-Cheng Ho, Ming Tatt Lee

**Affiliations:** 1 Faculty of Pharmaceutical Sciences, UCSI University, Kuala Lumpur 56000, Malaysia; 2 Department of Biomedical Sciences, Faculty of Medicine and Health Sciences, Universiti Putra Malaysia, Serdang 43400, Malaysia; 3 School of Medicine, College of Medicine, I-Shou University, Kaohsiung City 82445, Taiwan

**Keywords:** antioxidant, vitamin E, estrogen, bone health, osteoporosis, perimenopause, tocotrienols

## Abstract

Osteoporosis, or bone loss, is a disease that affects many women globally. As life expectancy increases, the risk of osteoporosis in women also increases, too, and this will create a burden on the healthcare and economic sectors of a country. Osteoporosis was once thought to be a disease that would occur only after menopause. However, many studies have shown that osteoporosis may develop even in the perimenopausal stage. Due to the erratic levels of estrogen and progesterone during the perimenopausal stage, studies suggest that women are exposed to the risk of developing osteoporosis even at this stage. The erratic hormonal changes result in the production of proinflammatory mediators and cause oxidative stress, which leads to the progressive loss of bone-building activities. Tocotrienols, members of vitamin E, have many health-promoting properties. Due to their powerful anti-oxidative and anti-inflammatory properties, tocotrienols have shown positive anti-osteoporotic properties in post-menopausal studies. Hence, we propose here that tocotrienols could also possibly alleviate perimenopausal osteoporosis by discussing in this review the connection between inflammatory mediators produced during perimenopause and the risk of osteoporosis. Tocotrienols could potentially be an anti-osteoporotic agent, but due to their low bioavailability, they have not been as effective as they could be. Several approaches have been evaluated to overcome this issue, as presented in this review. As the anti-osteoporotic effects of tocotrienols were mostly studied in post-menopausal models, we hope that this review could pave the way for more research to be done to evaluate their effect on peri-menopausal models so as to reduce the risk of osteoporosis from an earlier stage.

## 1. Introduction

Osteoporosis is a type of systemic skeletal disorder characterized by a reduced amount of low bone mass and microarchitectural deterioration of bone tissue. It is also one of the major causes of fractures in peri- and postmenopausal women [1]. Metabolic changes due to menopause are associated with chronic, low-grade inflammation and oxidative stress, which may cause osteoporosis, as both are contributors to bone loss [2]. Progressive bone loss and fractures due to osteoporosis are emerging public health problems in a world experiencing a rapid increase in peri- and postmenopausal women. An epidemiologic study showed that approximately 46.4% of women aged between 50 and 84 years are classified as having osteoporosis in 27 countries in the European Union, and the main risk factor for bone loss in midlife women was identified as menopause [3]. Although the risk of developing osteoporosis is more pronounced in postmenopausal women, there is evidence that indicates that bone loss actually began even before this stage. The perimenopausal stage or the menstrual transition period is defined as the stage before complete cessation of menstruation. Studies have shown that bone loss is significantly observed in women in the late phase of perimenopause and continued to early post menopause [4]. Currently, the first line of treatment for osteoporosis is calcium and vitamin D supplementation, which are essential elements for the maintenance of bone health. Calcium can be absorbed from food and taken as a supplement in tablet form. Furthermore, several kinds of drugs can also prevent osteoporosis by blocking bone reabsorption (e.g., bisphosphonates, denosumab), stimulating bone formation, or both (e.g., teriparatide, abaloparatide). Menopausal hormone therapy is also a well-established prophylactic measure that reduces the frequency of osteoporotic fractures, as estrogen can prevent bone loss in postmenopausal women, but prolonged use of estrogen may sometimes be ineffective and commonly associated with side effects such as mastalgia, colporrhagia, cardiovascular events, thromboembolic disease, and stroke and might even increase the risk of developing cancer, such as mammary cancer and endometrial carcinoma [1]. 

**Figure 1:**
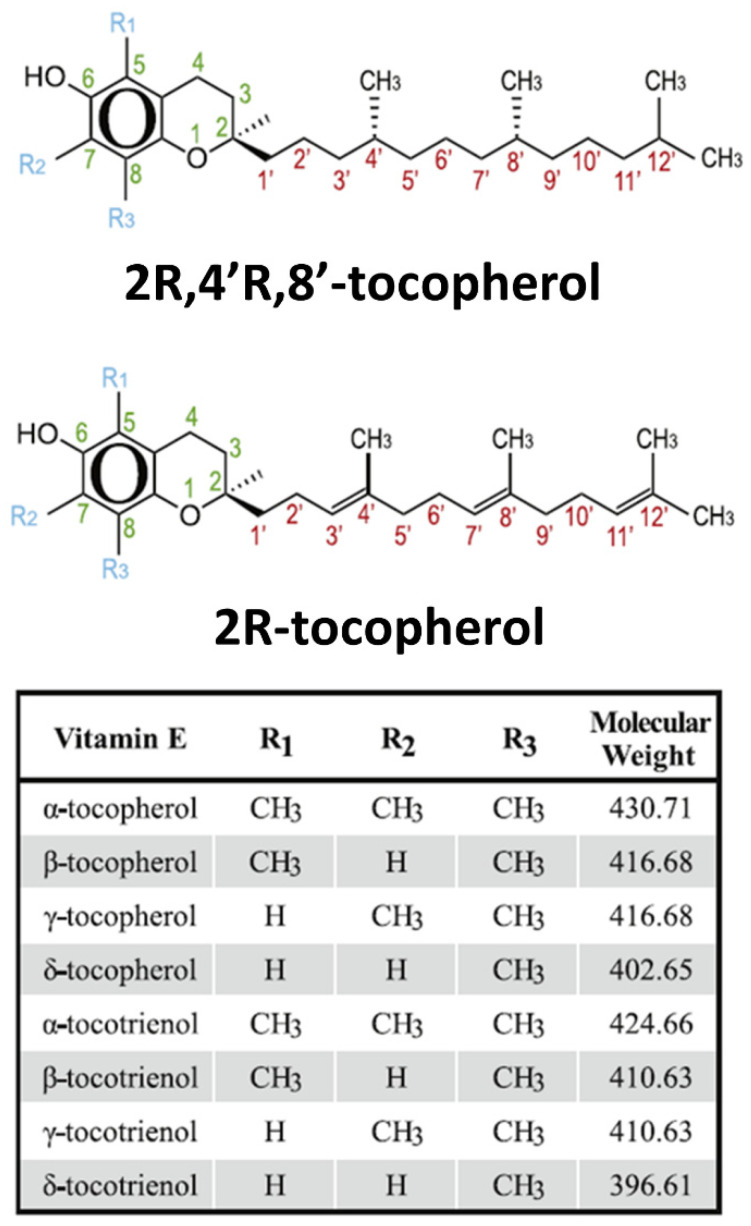
Molecular structure of tocotrienols adapted and slightly modified from Peh et al. (2016) [8]. *Reprinted/adapted with permission from [8]*.

Tocotrienols have been proposed as a potentially preventive measure for osteoporosis given the plausibility of its various biological functions in influencing the bone loss associated with the condition. Tocotrienols have multiple properties, including cardiovascular health protection, anti-cancers, neuroprotection and cognitive function, immune modulation, skin protection, bone protection, as well as potent antioxidant and anti-inflammatory characteristics [5]. Several of these roles offer a theoretical rationale for the benefits of the treatment of perimenopause-associated osteoporosis. A summary on the properties of tocotrienols is presented in Table 1, compiled from Ranasinghe et al. (2022) [6] and Wong and Radhakrishnan (2012) [7]. The molecular structures of tocotrienols are shown in Figure 1.

## 2. Tocotrienols’ Antioxidative Effects in Alleviating Osteoporosis

All vitamin E constituents exert antioxidant effect mainly via scavenging lipid peroxyl radicals, thus inhibiting the propagation chain of lipid peroxidation. As all vitamin E constituents possess phenolic moiety, they are all considered to have antioxidant capabilities [9]. Tocotrienols are minor forms of naturally occurring vitamin E and have a chroman ring as well as an unsaturated farnesyl side-chain with analogues of α-, β-, γ-, and δ-type [10]. Tocotrienols have far superior antioxidant effect when compared to tocopherols [11]. 

As noted by Serbinova et al., the antioxidant effect of tocotrienols is better compared to tocopherols, whereby the scavenging peroxyl radicals’ activity of α-tocotrienol was 1.5-fold higher in liposomes compared to α-tocopherol [12]. In addition, α-tocotrienol was also found to be 6.5 times better in protecting cytochrome P-450 against oxidative damage. Several factors could have contributed to these observations; α-tocotrienol is more uniformly distributed in the membrane bilayer, and it has a stronger disordering effect than its tocopherol counterpart. Apart from that, the chromanoxyl radical of α-tocotrienol was reported to be recycled more rapidly in membrane and lipoproteins, which correlates with the higher inhibition of lipid peroxidation as compared to α-tocopherol [13]. Hsieh et al.’s chemo-preventive study found that tocotrienols were able to alter the redox status of MCF-7 and MDA-MB-231 breast cancer cells by selectively increasing the basal levels of antioxidants (anti-thioredoxin) in MDA-MB-231 cells, increasing glutathione peroxidase (Gpx) expression (by δ-tocotrienol) and catalase (CAT) activity [14]. Both Gpx and CAT play important roles in the cellular defenses in the decomposition of hydrogen peroxide (H_2_O_2_). Furthermore, they also found a reduction in Kelch-like ECH-associated protein 1 (KEAP1) and increased nuclear factor erythroid-2-related factor (NRF2) in MDA-MB-231 cells, which could have led to the increased expression of the antioxidative enzymes [14].

Oxidative stress, as defined by the imbalances between reactive oxidative species (ROS) and antioxidants, has been implicated in osteoporosis. It may promote differentiation of osteoclasts and bone resorption, and in peri- and post-menopausal osteoporosis, increased oxidative stress during bone remodeling is correlated with the reduction of estrogen levels. A marked decrease of plasma antioxidants was also reported in both aged or osteoporotic rats and human subjects. However, the effects of oxidative stress could be reversed by antioxidants [15]. Numerous studies have been conducted on the antioxidative effects of tocotrienols on bone health. These studies range from in vitro and in vivo to human and have been comprehensively covered by Shen et al. [16] and Wong et al. [17]. Shen et al. illustratively proposed that tocotrienols’ protective effect on bone health lies in their antioxidant capability, as tocotrienols could suppress ROS production. Reduction of ROS reduces oxidative stress, which results in increased bone formation and mineralization, decreased bone resorption and erosion, as well as subsequently increased BMD and bone strength [16]. In vitro studies have demonstrated that tocotrienols are able to stimulate osteoblastogenesis and suppress osteoclastogenesis in protecting bone health. In a study using an H_2_O_2_-induced osteoblasts model, treatment of γ-tocotrienol (1 μM) for 24 h showed protective effect on osteoblasts from the harmful effects of H_2_O_2_. However, at 290 μM γ-tocotrienol, a cytotoxic effect on osteoblasts was observed. Another study looking at the effects of α-, δ-, and γ-tocotrienols on preosteoclasts cells showed reduced tartrate-resistant acid phosphatase-positive (TRAP^+^) osteoclast formation and reduced calcium phosphate resorption (δ- and γ-tocotrienols-treated cells) compared to the non-treated control group of cells [16]. Animal studies revolving around this aspect are extensive and cover both female ovariectomized and male rodent models. In ovariectomized models, short-term and long-term treatments with tocotrienols successfully increased femur bone mineral density, bone formation, and strength. Furthermore, the mRNA expression of osteocalcin, bone morphogenetic protein 2 (BMP-2), RUNX family transcription factor 2 (RUNX-2), and vascular endothelial growth factor-alpha (VEGF-α) of tibia were reported to be increased following treatments with tocotrienols [16,17]. Apart from that, a randomized, double-blinded, placebo-controlled trial evaluated the effects of 12-week γ- and δ-tocotrienols supplementation on the levels of receptor activator of nuclear kappa-B ligand (RANKL), osteoprotegerin (OPG), bone markers bone alkaline phosphatase (BALP), and N-terminal telopeptide (NTX) as well as oxidative stress biomarkers (8-hydroxy-2′-deoxyguanosine, 8-OHdG) in postmenopausal women with osteopenia, also showing positive results. There was a significant increase in the BALP/NTX ratio but a decrease in the RANKL/OPG ratio and 8-OHdG concentrations in postmenopausal women receiving tocotrienols supplementation relative to the placebo group [18]. An in vitro study that evaluated the effects γ-tocotrienol on lipid peroxidation, antioxidant enzymes activities, and apoptosis of osteoblasts exposed to H_2_O_2_ found that γ-tocotrienol could prevent malondialdehyde (MDA) elevation, reduce osteoblast apoptosis, and increase SOD, GPx, and CAT activities [19]. Tocotrienols, rice protein, melatonin, and other natural compounds have also been shown to reduce ROS by inducing heme oxygenase-1 (HO-1) activity, which may potentially reverse the damaging effects of oxidative stress [20,21,22]. 

## 3. Tocotrienols’ Anti-Inflammatory Effects in Alleviating Osteoporosis

**Figure 2:**
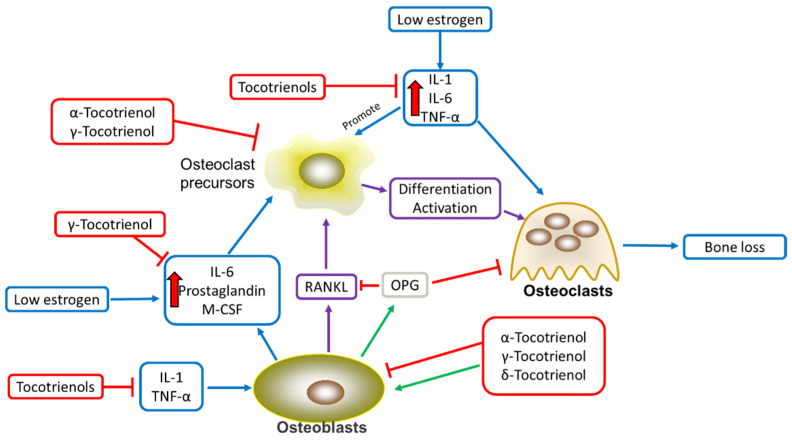
The effects of inflammatory mediators on bone cells and bone remodeling and the countereffect of tocotrienols.

Tocotrienols also acted as an anti-inflammatory agent in preventing bone loss by reducing the production of proinflammatory cytokines and eventually inflammation. In a lipopolysaccharide (LPS)-induced inflammation rat model, δ-tocotrienol was found effective in decreasing LPS-induced transcription and expression of TNF-α, interleukin-1 beta (IL-1β), IL-6, and inducible nitric oxide synthase (iNOS) at low concentrations. Further in vitro evaluation found that tocotrienols inhibited the LPS-stimulated secretion of inflammatory factors in RAW 264.7 macrophages through inhibition of the chymotrypsin-like, trypsin-like, and post-glutamase activities [23]. In human studies, tocotrienols have been reported to inhibit monocyte-endothelial cell adhesion, reduce endothelial expression of adhesion molecules, and adhere to monocytes [24], and it is more potent in inhibiting adhesion molecule expression and monocytic cell adherence [25]. In addition, the anti-inflammatory effect of tocotrienols was also demonstrated by Noguchi et al., and they found that tocotrienols were 10 times more efficient in reducing VCAM-1 expression and adhesion of THP-1 cells to HUVECs than tocopherols [26]. Further research also showed that α- and δ-tocotrienols can suppress osteoclastogenesis by inhibiting inflammatory cytokines, with the latter exhibiting more potent inhibition on osteoclast formation and activity than the former [27]. Besides that, γ- and δ-tocotrienols can enhance the osteogenic activity of murine osteoblasts by promoting the expression of bone formation-related genes as well as increasing the expression of osterix, collagen type 1 alpha 1, alkaline phosphatase (ALP), and osteocalcin (OCN), resulting in increased formation of collagen fibrils and mineralization of the extracellular matrix [28]. Figure 2 depicts the effects of elevated inflammatory mediators during the perimenopausal stage and the protective effects of tocotrienols.

Perimenopause is defined by a period of generally low estrogen levels, which increases the production of inflammatory mediators such as IL-1, IL-6, and TNF-α. IL-1 has both direct and indirect effects on bone resorption, as it acts directly on osteoclasts and indirectly by stimulating RANKL production. The increased activity of RANKL will in turn stimulate osteoclastogenesis. IL-1 also increases synthesis of prostaglandin, which may play a role in IL-1′s resorptive activity. IL-6 on the other hand regulates osteoclast progenitor cell differentiation into mature osteoclasts, directly stimulating both RANKL and osteoprotegerin (OPG) mRNA production and the production of prostaglandins [29]. In addition, TNF-α can strengthen osteoclasts formation by enhancing stromal cell production of RANKL and macrophage colony stimulating factor (MCSF) as well as the responsiveness of osteoclasts precursors to RANKL [29]. The effect of TNF-α on osteoclastogenesis can be enhanced by IL-1, whereby it increases RANKL expression in bone marrow stromal cells and directly promotes osteoclasts differentiation [30]. TNF-α also can promote the fusion of monocytes into osteoclasts. At the same time, it inhibits the differentiation of osteoblasts from progenitor cells [31]. Other inflammatory mediators, such as IL-1, CRP, and TNF-α, were also found to be slightly elevated in late perimenopause [32]. The increase of these inflammatory mediators in peripheral blood during the perimenopausal stage will undoubtedly result in the decrease of the OPG/RANKL ratio, promoting the differentiation of osteoclasts and finally leading to the increase of bone loss.

## 4. Inflammatory Mediators and Reactive Oxygen Species (ROS)

TNF-α, a pro-inflammatory cytokine, is produced by monocytes, macrophages, and osteoblasts [33]. TNF-α is linked to systemic inflammatory responses and is expressed during the acute inflammatory phase [34]. ROS plays a significant role in the pathogenesis of osteoporosis, and the formation of ROS is an inescapable consequence of life in an oxygen-rich environment. Mitochondria are the primary intracellular organelle responsible for ROS production [35], and the electron transport chain (ETC) complex I and ETC complex III produce superoxide ion (O_2_^−^) during electron transfer process [36,37]. TNF-α can increase ROS levels in mitochondria by inhibiting the electron transport chain and altering membrane permeability [37,38]. ROS also causes lipid, DNA, and protein oxidation [39]. A negative correlation has been noted between ROS production and bone-formation rate in osteoporosis, and this was coupled with reduced thiol antioxidant defenses in a study by Almeida et al. [40]. In addition, several studies have reported numerous changes such as increase in total plasma oxidant status, lipid oxidation, and SOD and decrease in catalase and glutathione peroxidase activity. These changes are linked to decreased bone mineral density in osteoporotic subjects, summarized by Wauquier et al. [41]. Excess ROS can induce osteoblast and osteocyte apoptosis as well as suppression of bone formation while enhancing osteoclastogenesis and bone resorption and degradation [42]. These happen with an increase in RANKL production, which causes hematopoietic precursors to differentiate into multinucleated osteoclasts [41]. 

O_2_^−^ that are formed in the mitochondria are converted into hydrogen peroxide (H_2_O_2_), which has the potential to suppress osteoblast cells differentiation [41]. In vitro, H_2_O_2_-induced oxidative stress can suppress the osteoblastic differentiation process of primary rabbit bone marrow stromal cells and calvarial osteoblasts via activation of the ERK-dependent NF-κB signaling pathway [42]. Clinical trials also show that ROS can increase the RANKL/OPG ratio, which can lead to bone loss and osteoporosis in the end [18]. High levels of H_2_O_2_ and ROS were reported in peri- and post-menopausal women, and this increased their risk to osteoporosis [43,44,45].

**Figure 3:**
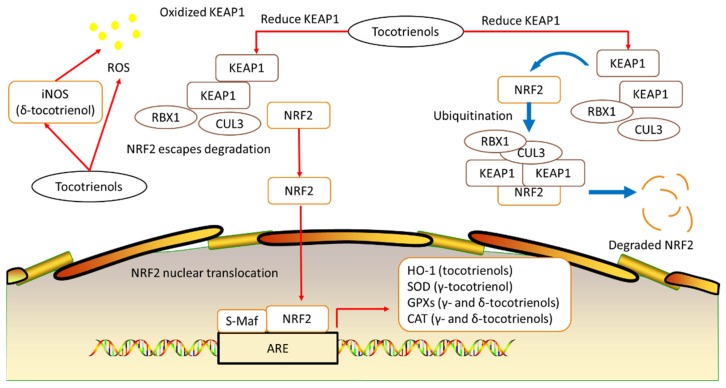
NRF2-KEAP1-AREs signaling pathway and the effects of tocotrienols.

To reduce the production of ROS, mitochondria produce several protective antioxidant enzymes such as superoxide dismutase (SOD), CAT, heme oxygenase-1 (HO-1), glutathione peroxidase 1 (GPx 1), and glutathione peroxidase 2 (GPx 2). As ROS could stimulate the differentiation of osteoclasts, and oxidative stress increases bone resorption in osteoporosis; Ke et al. (2015) suggested that the induction of antioxidant enzymes could possibly reverse the damaging effects of oxidative stress [22]. The transcription of these antioxidants is markedly regulated by nuclear respiratory factor 2 (NRF2). In basal conditions, Kelch-like ECH-associated protein 1 (KEAP1) recruits NRF2 into the Cul3-containing E3 ubiquitin ligase complex, which ubiquitylates NRF2 and leads to its degradation. In contrast, oxidative stress triggers the activation of NRF2 by inhibiting E3 ubiquitin ligase activity, resulting in dissociation between NRF2 and KEAP1. Next, Nrf2 translocates into the nucleus, and the heterodimerization of NRF2 with small Maf proteins in antioxidant response elements (AREs), a conserved gene sequence in promoter regions of target genes, activates transcription of SOD, CAT, HO-1, GPxs, and so on [46]. As mentioned, researchers have reported that NRF2 activators such as tocotrienols, rice protein, melatonin, and some other natural compounds could attenuate ROS via the NRF2-KEAP1-ARE pathway [20,21]. Research showed no evidence that the antioxidant enzyme activity of healthy premenopausal women is lower than that of women of childbearing age [47,48]. On the other hand, in both perimenopause and postmenopausal stages, the production and activities of SOD, CAT, HO-1, and GPxs are reduced to varying degrees. Ogunro et al. reported that the activities of GPxs, SOD, and CAT were obviously reduced in perimenopause and post-menopause phases compared to premenopausal phase, and these declines were negatively correlated with high concentrations of FSH and LH during this period [49]. Besides the activities, the synthesis of CAT and GPxs was also reduced in both peri- and post-menopausal phases, and these reductions could be reversed by hormone replacement therapy [50,51,52] (Figure 3).

## 5. Improving Tocotrienols’ Bioavailability for the Future of Anti-Osteoporotic Use

As perimenopausal bone loss could be attributed to proinflammatory mediators and the production of ROS leading to oxidative stress, the anti-oxidative and anti-inflammatory properties of tocotrienols may be beneficial in alleviating perimenopausal bone loss, too (similar to its beneficial effects seen in post-menopausal osteoporosis). However, tocotrienols have lower bioavailability compared to their isomer, tocopherols. Low plasma concentrations of tocotrienols have been reported in which the absorption of tocotrienols was found to be negligible when administered via the intraperitoneal and intramuscular routes. The low bioavailability can be attributed to the lower affinity of α-tocopherol transfer protein for tocotrienols as compared to tocopherols [53]. When given via the oral route in rats, incomplete absorption of tocotrienols was observed. Thus, various research efforts have been undertaken to formulate tocotrienols in solubility-enhanced delivery systems. Apart from that, tocotrienols have also been formulated with cyclodextrin, Tween 80, and in the form of nano-carriers (for intravenous administration) with improved absorption and higher plasma concentration profiles in rats [53]. 

Due to the high potential therapeutic effects of tocotrienols on bone loss but low bioavailability, Ibrahim et al. investigated the potential of combining synthetic and biodegradable polymers with tocotrienols and delivering them in a controlled manner [15]. A formulation of nanocarrier polylactic-co-glycolic acid (PLGA) and tocotrienols was injected intra-osseously once into the bones of ovariectomized rats and compared to rats given daily oral tocotrienols for 8 weeks. Their results revealed similar potency; significant improvements were noted in lower bone markers, higher bone mineral content, higher trabecular separation and connectivity density, and better bone strength [15]. With these results, a rapid and safer method was born, and it will be beneficial to patients for whom venous access is difficult as well as those in coma or immobile, with fewer compliance issues and fewer side effects [53]. In a similar manner of utilizing the concept of a controlled drug-delivery system, the same research group also showed that the combination of lovastatin and tocotrienols improved the fracture healing of ovariectomized osteoporotic rats [54]. Compared to the ovariectomized rats that were treated with only lovastatin and tocotrienols, the combination of lovastatin and tocotrienols showed higher callus volume and callus strength, while the single-treatment groups only showed improvement in callus strength. Statin, which also has low bioavailability like tocotrienols, has shown promising results in increasing bone formation, but high doses are needed. However, high doses of statin cause liver failure, kidney disease, and rhabdomyolysis [54]. Thus, by combining two drugs with low bioavailability but with great bone-healing properties, the issues of low bioavailability and side effects were overcome by a controlled drug-delivery system. 

Annatto seed, with the richest source of δ-tocotrienol (about 90%), has been reported to prevent bone loss in ovariectomy-induced animal models of bone loss, but it lacked evidence in vivo studies to support its preventative nature in a model that mimics clinical scenarios [55]. Thus, Mohamad et al. investigated the effectivity of a self-emulsifying drug-delivery system (SEDDS) with the tocotrienols extracted from annatto seeds [55]. The system is made of surfactant, co-solvent, and oils, and it demonstrated improvement in the colloidal dispersion of tocotrienols. A nanoemulsion of the tocotrienols was formed in the gut, and together with gastrointestinal fluid, a larger surface area for unloading of molecules was created. It also increases drug partitioning through the intestinal wall. In this study, the annatto tocotrienols formulated with SEDDS were evaluated in terms of their effect on bone parameters and oxidative stress markers of an ovariectomized animal model of bone loss. After eight weeks of treatment, both annatto (60 mg/kg body weight)-treated rats with and without SEDDS showed improvements in the cortical bone thickness, preserved bone calcium content, increased bone biomechanical strength, and increased antioxidant enzyme activities in comparison to the non-treated ovariectomized group. The SEDDS-formulated-annatto-treated rats showed a higher amount of circulating δ-tocotrienol compared to the non-SEDDS-formulated ones. Despite their similarities in the above-mentioned parameters, SEDDS-formulated-annatto-treated rats fared better in the trabecular microstructure, bone stiffness, and MDA levels (lower) analyses [55]. In another study, Mohamad et al. found supportive results regarding the effects of SEDDS-formulated-annatto treatment [56]. In this study, the annatto tocotrienols were formulated in a self-emulsifying system. Their self-emulsified annatto tocotrienols (SEAT) showed similar potency in improving the number of osteoclasts and trabecular mineralization rate. SEAT was found to be superior in the bone-formation rate and significantly reduced the RANKL/OPG ratio compared to unformulated annatto tocotrienols. In conclusion, Mohamad et al. proved that their self-emulsifying systems increased the bioavailability of annatto tocotrienols, improving the skeletal therapeutic effects and skeletal anabolic properties of annatto tocotrienols in rats deficient in estrogen [56].

As vitamin E, which includes tocotrienols and tocopherols, cannot be produced by our body, it must be supplemented by other means. Vitamin E is generally incorporated into chylomicrons from the intestinal mucosa. With the facilitation of α-tocopherol transport protein in the liver, vitamin E is then be carried in plasma lipoproteins throughout the circulatory system [8]. Hence, incorporating vitamin E, specifically tocotrienols, into one’s daily diet could be beneficial. Palm oil is another example of natural source that is rich in tocotrienols. Present within the palm oil–tocotrienol-rich fraction (TRF) are 18–22% of tocopherols and 78–82% of tocotrienols, in which are found the α-, γ-, and δ-isoforms of tocotrienols. A diet high in tocotrienols has been shown in studies to slow the ageing process and its effects as well as reduce the incidence of several chronic diseases. In terms of being an anti-osteoporotic candidate, tocotrienols from palm oil have also shown promising results. The cumulative anti-osteoporosis benefits of palm oil tocotrienols include a reduction in bone loss in postmenopausal female rats, improved bone strength and fracture resistance, and increased bone density. Palm oil tocotrienols also preserved the levels of cytokines involved in osteoclast differentiation and improved the cellular and dynamic properties of rat bone [57]. In addition, tocotrienols also strengthen young adults’ peak bone mass, which could be an indication of preventative effect of tocotrienols [58].

## 6. Conclusions

Due to the decline of estrogen and its protective effects and oxidative stress, osteoporosis is certainly a risk for women at menopause. With evidence indicating that women at the perimenopausal stage are similarly at risk of developing osteoporosis, it is indeed a matter that has to be resolved to reduce the burden on them and also the country’s healthcare fraternity as a whole. As tocotrienols have proven to be an effective source in reversing bone loss, we believe that it could be introduced into the healthcare system as an additional measure to treat osteoporosis. More research is needed to address the issue of low bioavailability and the benefit of tocotrienols in the perimenopausal stage. 

## Figures and Tables

**Table 1:** The multifaceted properties of tocotrienols.

Property	Disease/Model	Tocotrienol	Combine with	Effect(s)
Anti-cancer	Bladder and pancreas cancer	Gamma- and delta-tocotrienols	Gemcitabine	Induced p21 and p27 Suppressed MMP-9, COX-1, Ki-67, Cyclin-D1, Bax, Bcl-1, Bcl-2, Bcl-xL, and Mcl-1 Activated caspase-3, PARP cleavage Downregulated STAT3 and NF-κB/65 pathways
	Liver cancer		Paclitaxel Doxorubicin	Inhibited NF-κB, JakStat-3/6. STAT-3, 5-LOX-induced eicosanoids, PGE_2_, and LTB_4_
	Brain cancer		Jerantinine A	Induced apoptosis, Fas and p53, and G0/G1 cell cycle arrest
	Gastric and colorectal cancer		Capecitabine	Downregulated survivin, cIAP-1, cIAL-2, Cyclin D1, c-myc, MMP-p, VEGF, ICAM-1, and CXCR4 by inhibiting NFκB/p65 pathway
	Prostate cancer		Docetaxel	Modulated pro-survival Id-1, Id-3, EGF-R, JNK, NF-κB, sphingolipids, eicosanoids, and Ang-1/Tie-2 pathway 2
		Tocotrienols		Inhibited cell growth in PC3 and LNCaP prostate cancer cells
		Gamma-tocotrienol		Suppressed NF-κB, EGFR, and Id family proteins (Id1 and Id3)
	Oral cancer	Gamma- and delta-tocotrienols		Inhibited docetaxel-induced NF-κB/p65 pathway
	Breast cancer			Activated caspases, EGF-R, and Id-1 and suppressed NF-κB
	Breast cancer		Tamoxifen	Activated estrogen-responsive genes, caspase-3, and apoptosis
		TRF–palm oil		Prevented chemically induced mammary tumorigenesis
	Human breast cancer cells	Tocotrienols		Effective apoptotic inducers
	Mammary cancer cells	Tocotrienols		Induced apoptosis by activating caspase-8 signaling pathway and disrupted mitochondrial function
	Breast cancer cells	Tocotrienols		Induced cell death on proteins with inhibitory effects on cell growth and differentiation MM-1 and interferon-inducible protein 9–27
	Breast cancer cells	Gamma-tocotrienol		Induced cell death by disrupting Id1, a key cancer-promoting protein
	Cervical cancer	Alpha- and gamma-tocotrienols		Inhibited HeLa cell proliferation by upregulating IL-6 and downregulating cyclin D3, p16, and CDK6 expression in the cell-cycle signaling pathway
	Mesothelioma	Gamma- and delta-tocotrienols	Cisplatin	Suppressed PI3K/Akt pathway
	Melanoma		Statin	Inhibited mevalonate pathway Inactivated Ras
Antioxidant	Primary hippocampal neurons	Gamma-tocotrienol		Provided neuroprotection by upregulating the Bcl-xL family of proteins, which induces anti-apoptosis and maintain optimum neurotransmission
		Alpha-tocotrienol		Provided neuroprotection to glutamate-induced hippocampal neurons by preventing the loss of mitochondrial membrane potential
	+SA mammary tumor epithelial cells	Gamma-tocotrienol		Provided anti-apoptotic effects by increasing PARP cleavage and activation-mediated protein kinase-like ER kinase/eukaryotic translational initiation factor/activating transcription factor (PERK/eIF2alpha/ATF-4) pathway, a marker of ER stress response
	Osteoblasts exposed to hydrogen peroxide	Gamma-tocotrienol		Reduced production of malondialdehyde and free radical release and increased antioxidant status in cancerous patients in a dose-dependent manner Prevented the reduction of SOD, catalase, and Gpx activity at lower concentrations
	Experimental diabetic rats	TRF		Increased SOD activity and vitamin C levels, reduced lipid peroxidation in the thoracic aorta homogenates, and reduced vascular smooth muscle cell proliferation and degeneration
	UV-radiation-induced oxidative stress in murine skin	TRF		Inhibited oxidative stress
	Diabetic animals	TRF		Improved wound-healing mechanisms
	High-fat-diet- and streptozotocin-induced diabetes	TRF		Reduced skeletal muscle-related protein levels (4-hydroxynonenal, protein carbonyls, Nrf2, and HO-1)
Anti-inflammatory	LPS- and SFA-stimulated NLRP-3 inflammasome activation in BMDM in vitro	Gamma-tocotrienol		Repressed NLRP3 inflammasome activation by downregulating arachidonic acid metabolism, diacylglycerol, prostaglandin release, and COX-2 activation
	NLRP-3 inflammasome activation in macrophages in vitro	Annatto-seed-derived delta-tocotrienol		Inhibited the priming of the inflammasome by suppressing the NF-κB pathway and ROS production
	Chronic inflammation mimetic type 2-diabetes (leptin receptor knockout mice)	Gamma-tocotrienol		Attenuated the NLRP3-inflammasome activity by downregulating A20-induced TNF-α interacting protein 3-activated NF-κB signaling cascade and suppressed caspase-1 cleavage by AMP-activated protein kinase autophagy
		TRF		Inhibited iNOS and COX-2 production and NF-κB expression
Cardioprotective (i) Lipid-lowering effects		Delta- and gamma-tocotrienols		Reduced triglyceride synthesis and transport
		Tocotrienols		Reduced synthesis and increased degradation of HMG-CoA reductase Influenced mevalonate pathway in mammalian cells by post-transcriptional suppression of HMG-CoA reductase
		Tocotrienols in chick diet		Lowered hepatic cholesterogenesis, serum total cholesterol, and low-density lipoprotein cholesterol with concomitant increase in lipogenic activity
(ii) Antiatherogenic effects		Rice bran tocotrienols		Reduced size of atherosclerotic lesions in mice
	Patients with high cholesterol	Tocotrienols		Reversal of arterial blockage of carotid artery
	Atherosclerosis-prone mice	Palm oil tocotrienols		Reduced formation of lesion through antioxidant-dependent and -independent mechanisms
(iii) Others		Tocotrienols		Provided protection against endothelial dysfunction and platelet aggregation Lowered blood pressure Improved arterial compliance
		Palm oil tocotrienols		Stabilized proteasomes
Neuroprotective		Palm oil alpha-tocotrienol		Attenuated both enzymatic and nonenzymatic mediators of arachidonic acid metabolism and neurodegeneration
		Alpha-tocotrienol		Reduced size of cerebral infarcts in mice
Anti-diabetic	Diabetic rats	TRF		Improved blood glucose, dyslipidemia, and oxidative stress
	Type 2 diabetes	Beta-tocotrienol	Total vitamin E	Reduced risk of type 2 diabetes mellitus
	Streptozotocin-induced diabetes	Tocotrienols-rich diet		Decreased advanced glycosylation end products in nondiabetic rats Improved glycemic control in rats with streptozotocin-induced diabetes
Anti-osteoporosis		Alpha-tocotrienol		Reduced proinflammatory effect of TNF-α, expression of RANKL in osteoblasts Inhibited the differentiation of IL-1 induced-osteoclast Reduced osteoclast formation
		Gamma-tocotrienol		Inhibited IL-1-mediated PGE_2_ and PGD_2_ production Inhibited COX-2 and 5-LOX from transforming arachidonic acid into an inflammatory mediator Increased transcription and expression of SOD, Gpx, and catalase Protected osteoblasts from harmful ROS damages Increased formation of collagen fibrils Increased mineralization of extracellular matrix Increased OPG and reduced RANKL and osteoclast formation
		Delta-tocotrienol		Reduced the expression of TNF-α, IL-1β, and IL-6 Inhibited 5-LOX from transforming arachidonic acid into an inflammatory mediator Reduced the expression of iNOS Increased the expression and activities of Gpxs and catalase Increased formation of collagen fibrils Increased mineralization of extracellular matrix Increased OPG and reduced RANKL and osteoclast formation
		Tocotrienols		Suppressed the synthesis of leukotrienes Reduced the levels of IL-1 and IL-6 Reduced KEAP1, promoting transcription of HO-1, Gpxs, and SOD Increased mRNA expression of osteocalcin, BMP-2, RUNX-2, and VEGF-α of tibia and many protective effects

## Data Availability

Not applicable.
